# Right heart function matters: prognostic value of sPAP, TAPSE and RV–PA coupling in a real-world cohort of TAVI patients

**DOI:** 10.1007/s12928-026-01257-3

**Published:** 2026-02-25

**Authors:** Elke Boxhammer, Sophie Aglas, Nikolaus Clodi, Michael Lichtenauer, Richard Rezar, Christian Dinges, Christina Granitz, Crispiana Cozowicz, Uta C. Hoppe, Matthias Hammerer

**Affiliations:** 1 Department of Internal Medicine II, Division of Cardiology, Paracelsus Medical University of Salzburg, 5020 Salzburg, Austria; 2 Department of Cardiovascular and Endovascular Surgery, Paracelsus Medical University of Salzburg, 5020 Salzburg, Austria; 3 Department of Anesthesiology, Perioperative Medicine and Critical Care Medicine, Paracelsus Medical University of Salzburg, 5020 Salzburg, Austria

**Keywords:** Right Heart Function, RV-PA Coupling, sPAP, TAPSE, TAVI

## Abstract

Right ventricular (RV) function is an established prognostic factor in structural heart disease, but is not routinely incorporated into pre-procedural risk assessment in patients undergoing transcatheter aortic valve implantation (TAVI). This study evaluated the prognostic impact of systolic pulmonary artery pressure (sPAP), tricuspid annular plane systolic excursion (TAPSE), and RV–pulmonary artery (PA) coupling (TAPSE/sPAP ratio) in a real-world TAVI population. This retrospective single-center study included 565 consecutive patients (mean age 82.1 ± 5.1 years; 48.7% male) undergoing transfemoral TAVI between 2016 and 2022. All patients received standardized pre-procedural echocardiographic assessment of sPAP, TAPSE, and TAPSE/sPAP. The primary endpoint was all-cause mortality over a mean follow-up of 47.1 ± 22.8 months. Impaired RV–PA coupling – defined as a TAPSE/sPAP < 0.55 mm/mmHg – was identified in 46.5% of patients, elevated sPAP (≥35 mmHg) in 61.2%, and reduced TAPSE (≤18 mm) in 22.3%. Both elevated sPAP and reduced TAPSE/sPAP were significantly associated with increased long-term mortality (p=0.004 and p<0.001, respectively), whereas TAPSE alone was not predictive (p=0.318). Subgroup and interaction analyses showed that the prognostic impact of sPAP and TAPSE/sPAP was greatest in patients aged ≥80 years, in males, and in those with preserved left ventricular ejection fraction and normal stroke volume index (SVi).RV afterload, as reflected by elevated sPAP and impaired RV–PA coupling, is a key driver of post-TAVI mortality, outperforming isolated RV systolic measurements. The TAPSE/sPAP ratio is a robust, integrative marker whose prognostic value is modulated by age, sex and left heart function. These findings support incorporating RV–PA coupling into routine pre-TAVI assessment to improve risk stratification and identify vulnerable patients before intervention

**Graphical Abstract:**

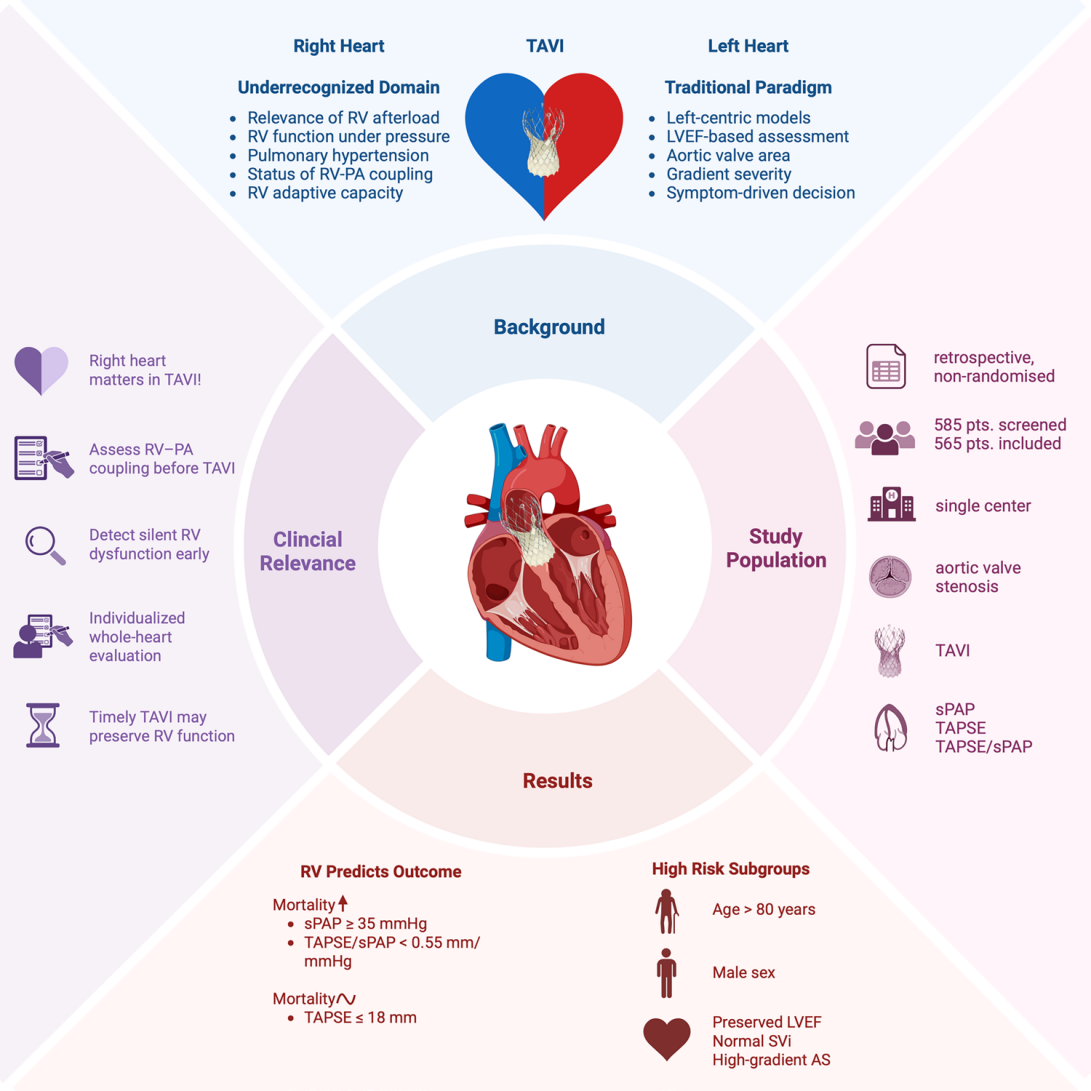

## Introduction

The right ventricle (RV) is an important determinant of outcome in patients exposed to chronic hemodynamic stress, including those undergoing transcatheter aortic valve implantation (TAVI) [1, 2]. In severe aortic stenosis, the clinical course is not determined solely by left ventricular (LV) pressure overload, but also by secondary changes within the pulmonary circulation and the right heart [3].

Chronically elevated LV filling pressures in aortic stenosis lead to increased left atrial and pulmonary venous pressures, which are transmitted into the pulmonary circulation and result in progressive RV afterload [4–6]. This pressure burden challenges the adaptive capacity of the RV and may substantially influence long-term outcomes after TAVI [5, 6].

Pre-procedural echocardiographic assessment of right heart function commonly relies on systolic pulmonary artery pressure (sPAP) as a measure of RV afterload, tricuspid annular plane systolic excursion (TAPSE) as an index of RV systolic function, and the TAPSE/sPAP ratio as an integrative marker of RV–pulmonary arterial (PA) coupling [7, 8]. While these parameters are readily available and widely used in clinical practice, their prognostic relevance may differ depending on patient phenotype and hemodynamic context.

Several studies have demonstrated an association between elevated sPAP, impaired RV function, and adverse outcomes in patients undergoing TAVI [3, 9–18]. Impaired RV–PA coupling, in particular, has been consistently associated with increased mortality across different patient populations and risk profiles [12–18].

Despite this growing body of evidence, it remains unclear in which clinical and hemodynamic settings right heart parameters provide incremental prognostic information beyond established risk factors, and whether their impact differs according to age, sex, left ventricular function, stroke volume, or aortic stenosis phenotype. Therefore, the present study aimed to evaluate the prognostic value of sPAP, TAPSE, and RV–PA coupling in a contemporary real-world TAVI cohort and to explore their relevance across clinically meaningful subgroups defined by age, sex, left ventricular ejection fraction (LVEF), stroke volume index, and aortic stenosis phenotype.

## Material & methods

### Study cohort

This single-center retrospective study included 585 consecutive patients undergoing transfemoral TAVI between January 2016 and December 2022. Patients were evaluated by an interdisciplinary heart team according to institutional standards. To minimize confounding, 20 patients with non–valvular causes of pulmonary hypertension or advanced pulmonary or systemic disease were excluded, resulting in a final cohort of 565 patients. All procedures were performed for native aortic valve stenosis; valve-in-valve interventions were not included in this study.

### Ethical considerations

The study was approved by the Ethics Committee of the State of Salzburg (EK-Nr. 1082/2024). Owing to its retrospective design, the requirement for written informed consent was waived.

### Data acquisition and management

Clinical data were obtained from the institutional electronic health records and archival databases, including medical history, procedural reports, and echocardiographic imaging.

### Transthoracic echocardiography

All patients underwent standardized transthoracic echocardiographic (TTE) assessment within 1 to 4 weeks prior to TAVI. Echocardiographic examinations were carried out using iE33 or Epiq 5 ultrasound systems (Philips Healthcare, Hamburg, Germany) by experienced cardiologists (≥ 4 years of dedicated echocardiographic practice) in compliance with contemporary ESC guidelines [19, 20].

LVEF was quantified using Simpson’s biplane method. Systolic pulmonary artery pressure (sPAP) was estimated from the peak tricuspid regurgitation (TR) velocity using the simplified Bernoulli equation, with right atrial pressure derived from inferior vena cava diameter and inspiratory collapsibility [21]. TR Doppler signals were obtained from multiple acoustic windows, and the highest-quality, clearly traceable spectral envelope was used for sPAP calculation. Pulmonary hypertension was defined as sPAP ≥ 35 mmHg according to current recommendations [22].

RV systolic function was assessed using tricuspid annular plane systolic excursion (TAPSE), measured by M-mode in the RV-focused apical four-chamber view with alignment parallel to the direction of annular motion. RV–pulmonary arterial (RV–PA) coupling was calculated as the TAPSE/sPAP ratio. Impaired RV–PA coupling was defined as TAPSE/sPAP <0.55 mm/mmHg in accordance with prior literature [23]. A TAPSE value ≤18 mm was considered indicative of reduced RV systolic function.

Inter-observer variability was assessed in a randomly selected subset of echocardiographic studies. All examinations had been originally acquired and clinically reported by experienced echocardiographers as part of routine care. For the purpose of this study, sPAP and TAPSE were independently re-measured offline by two experienced echocardiographers who were blinded to clinical data and outcomes. Agreement between observers was evaluated using intraclass correlation coefficients (ICC; two-way random-effects model, absolute agreement). In addition, Bland–Altman analyses were performed to assess systematic bias and limits of agreement.

### TAVI Procedure

All patients underwent transfemoral TAVI using CoreValve™ Evolut™, CoreValve™ Evolut™ R, CoreValve™ Evolut™ PRO and CoreValve™ Evolut™ PRO+ self-expanding prosthetic valves (Medtronic Inc., Minneapolis, MN, USA). Valve sizing and procedural planning were guided by pre-interventional imaging, including TTE, contrast-enhanced computed tomography angiography (CTA), and, where indicated, transesophageal echocardiography (TEE). TAVI procedures were performed according to the instructions for use of the manufacturer.

### Outcome measures

The primary endpoint of the study was all-cause mortality, assessed from the date of TAVI to a maximum follow-up duration of 84 months. All-cause mortality was selected to avoid potential misclassification, as reliable adjudication of cardiac-specific causes of death or heart failure–related hospitalizations was not feasible in this retrospective cohort. Survival status was determined through a combination of institutional records, correspondence with primary care physicians, direct communication with family members, and review of official death certificates.

### Statistical analysis

Statistical analyses were performed using SPSS software (Version 25.0, SPSS Inc., Armonk, NY, USA) and R (Version 4.2.3, R Foundation for Statistical Computing, Vienna, Austria). Visualizations were carried out using SPSS and GraphPad Prism software (GraphPad Prism version 8.0.0, GraphPad Software, San Diego, CA, USA).

Variable distributions were evaluated with the Kolmogorov–Smirnov–Lilliefors test. Continuous variables with normal distribution are presented as mean ± SD and compared using the unpaired Student’s t-test. Non-normally distributed variables are presented as median (IQR) and compared using the Mann–Whitney U test. Categorical variables are reported as frequencies (%) and compared using the chi-squared test.

Kaplan–Meier analysis was used to compare long-term mortality between patients with and without right ventricular dysfunction, with differences assessed using the log-rank test.

Associations between right heart parameters and long-term mortality were assessed using univariate and multivariate Cox proportional hazards regression. Follow-up time was defined from the date of TAVI to death, with surviving patients right-censored. Hazard ratios (HR) with 95% confidence intervals (CI) were calculated. Subgroup analyses were performed for age, sex, LVEF, SVi and AS gradient. Multivariate models used the enter method without stepwise selection.

Interaction terms were added to assess effect modification (e.g., sPAP ≥ 35 mmHg × age ≥ 80 years, TAPSE ≤ 18 mm × LVEF ≥ 50%). The proportional hazards assumption was verified using Schoenfeld residuals, and multicollinearity was assessed.

A two-tailed p-value < 0.05 was considered statistically significant. Bonferroni correction was applied for predefined families of subgroup and interaction analyses.

## Results

### Inter-observer reproducibility of echocardiographic parameters

Inter-observer agreement was good for echocardiographic parameters of sPAP and TAPSE, with an ICC of 0.84 for sPAP and 0.91 for TAPSE. Bland–Altman analyses demonstrated no relevant systematic bias.

### Clinical profile stratified by right ventricular function and pulmonary pressure

The study cohort included 565 patients undergoing transfemoral TAVI. Elevated sPAP (≥35 mmHg) was present in 346 patients (61.2%), reduced TAPSE in 126 (22.3%), and impaired RV–PA coupling (TAPSE/sPAP < 0.55) in 263 (46.5%). The mean age was 82.1 ± 5.1 years, 62.5% were ≥ 80 years old, and 48.7% were male. Patients with reduced TAPSE or impaired RV–PA coupling were older than their counterparts (p = 0.014 and p = 0.005), whereas sex distribution was similar across groups.

Patients with elevated sPAP and impaired RV–PA coupling more frequently had advanced heart failure symptoms (NYHA ≥ III: 48.3% vs. 37.0% and 50.2% vs. 38.4%, respectively), higher rates of atrial fibrillation (all p < 0.001), and higher NT-proBNP levels compared with their reference groups.

Reduced TAPSE and impaired RV–PA coupling were associated with lower LVEF (47.6 ± 12.7% and 51.1 ± 10.7%, both p < 0.001), lower stroke volume index (41.0 ± 14.3 and 44.2 ± 16.2 ml/m^2^, both p ≤ 0.001), and more frequent moderate or greater mitral and tricuspid regurgitation (all p < 0.001).

Overall, patients with right heart dysfunction exhibited a higher comorbidity burden and surgical risk, including higher creatinine levels and higher EuroSCORE II (all p < 0.001; Table 1).

**Table 1:** Baseline characteristics of the overall cohort and subgroups defined by sPAP ≥ 35 mmHg, TAPSE ≤ 18 mm, and impaired RV–PA coupling (TAPSE/sPAP < 0.55 mm/mmHg)

	Total	sPAP ≥ 35 mmHg			TAPSE ≤ 18 mm			TAPSE/sPAP < 0.55 mm/mmHg		
		Yes	No	p	Yes	No	p	Yes	No	p
**No. (%)**										
Total	565 (100.0)	346 (61.2)	219 (38.8)	-	126 (22.3)	439 (77.7)	-	263 (46.5)	302 (53.5)	-
Sex (male)	275 (48.7)	173 (50.0)	102 (46.6)	0.448	66 (47.6)	209 (47.6)	0.333	132 (50.2)	143 (47.5)	0.530
NYHA ≥ III	248 (43.9)	167 (48.3)	81 (37.0)	**0.016**	57 (45.2)	191 (43.5)	0.643	132 (50.2)	116 (38.4)	**0.008**
Diabetes Mellitus	152 (26.9)	86 (24.9)	66 (30.1)	0.168	34 (27.0)	118 (26.9)	0.981	69 (26.2)	83 (27.5)	0.739
Arterial Hypertension	491 (86.9)	296 (85.5)	195 (89.0)	0.179	113 (89.7)	378 (86.1)	0.319	227 (86.3)	264 (87.4)	0.622
CHD CHD — 1 vessel CHD — 2 vessels CHD — 3 vessels	177 (31.3) 96 (17.0) 78 (13.8)	104 (30.1) 60 (17.3) 38 (11.0)	73 (33.3) 36 (16.4) 40 (18.3)	0.440 0.758 **0.016**	42 (33.3) 22 (17.5) 24 (19.0)	135 (30.8) 74 (16.9) 54 (12.3)	0.603 0.890 0.055	76 (28.9) 45 (17.1) 34 (12.9)	101 (33.4) 51 (16.9) 44 (14.6)	0.180 0.942 0.574
AF	206 (36.5)	154 (44.5)	52 (23.7)	**<0.001**	74 (58.7)	132 (30.1)	**<0.001**	139 (52.9)	67 (22.2)	**<0.001**
PAOD	50 (8.8)	31 (9.0)	19 (8.7)	0.908	13 (10.3)	37 (8.4)	0.510	23 (8.7)	27 (8.9)	0.935
COPD	64 (11.3)	43 (12.4)	21 (9.6)	0.300	16 (12.7)	48 (10.9)	0.582	36 (13.7)	28 (9.3)	0.098
MI — Prehistory	40 (7.1)	24 (6.9)	16 (7.3)	0.867	9 (7.1)	31 (70.6)	0.975	20 (7.6)	20 (6.6)	0.650
Malignancy — Prehistory	99 (17.5)	61 (17.6)	38 (17.4)	0.932	29 (23.0)	70 (15.9)	0.066	46 (17.5)	53 (17.5)	0.985
Stroke — Prehistory	48 (8.5)	31 (9.0)	17 (7.8)	0.619	12 (9.5)	36 (8.2)	0.639	22 (8.4)	26 (8.6)	0.917
LVEF ≤ 40 41 - 54 ≥ 55	70 (12.4) 119 (21.1) 376 (66.5)	49 (14.2) 80 (23.1) 217 (62.7)	21 (9.6) 39 (17.8) 159 (72.6)	0.108 0.131 **0.015**	34 (27.0) 33 (26.2) 59 (46.8)	26 (5.9) 86 (19.6) 317 (72.2)	**<0.001** 0.109 **<0.001**	45 (17.1) 66 (25.1) 152 (57.8)	25 (8.3) 53 (17.5) 224 (74.2)	**0.001** **0.028** **<0.001**
SVi ≥ 35 ml/m^2^	433 (76.6)	258 (74.7)	175 (79.9)	0.043	75 (59.5)	358 (81.5)	**<0.001**	206 (78.3)	227 (75.2)	**<0.001**
High Gradient AS	421 (74.5)	259 (74.8)	162 (74.0)	0.875	83 (65.9)	338 (77.0)	0.014	189 (71.9)	232 (76.8)	0.209
MR ≥ moderate	199 (35.2)	148 (42.8)	51 (23.3)	**<0.001**	53 (42.1)	146 (33.3)	0.088	127 (48.3)	72 (23.8)	**<0.001**
TR ≥ moderate	157 (27.8)	140 (40.5)	17 (7.8)	**<0.001**	54 (42.9)	103 (23.5)	**<0.001**	130 (49.4)	27 (8.9)	**<0.001**
**Mean ± SD**										
Age (years)	82.1 ± 5.1	82.3 ± 4.9	81.8 ± 5.4	0.257	83.1 ± 5.2	81.8 ± 5.1	**0.014**	82.8 ± 5.1	81.5 ± 5.1	0.005
Height (cm)	167.6 ± 8.7	167.6 ± 8.4	167.7 ± 9.0	0.854	167.6 ± 8.6	167.6 ± 8.7	0.913	167.5 ± 8.5	167.8 ± 8.9	0.690
Weight (kg)	72.9 ± 14.5	72.5 ± 8.4	73.7 ± 14.9	0.326	70.9 ± 13.3	73.5 ± 14.8	0.069	72.0 ± 14.7	73.7 ± 14.3	0.170
BMI (kg/m^2^)	25.9 ± 4.4	25.7 ± 4.5	26.1 ± 4.3	0.274	25.2 ± 4.1	26.1 ± 4.5	0.052	25.6 ± 4.5	26.1 ± 4.3	0.149
BSA (m^2^)	1.8 ± 0.2	1.8 ± 0.2	1.8 ± 0.2	0.419	1.8 ± 0.2	1.8 ± 0.2	0.150	1.8 ± 0.2	1.8 ± 0.2	0.157
LVEF (%)	53.0 ± 9.8	52.4 ± 10.1	53.8 ± 9.2	0.096	47.6 ± 12.7	54.5 ± 8.2	**<0.001**	51.1 ± 10.7	54.6 ± 8.7	**<0.001**
SVi (ml/m^2^)	46.7 ± 15.7	47.5 ± 14.9	46.2 ± 16.2	0.344	41.0 ± 14.3	48.3 ± 15.8	**<0.001**	44.2 ± 16.2	48.8 ± 15.1	**0.001**
LVEDD (cm)	4.6 ± 0.7	4.7 ± 0.7	4.6 ± 0.7	0.185	4.7 ± 0.7	4.6 ± 0.7	0.566	4.7 ± 0.7	4.6 ± 0.6	0.121
IVSd (mm)	13.4 ± 2.2	13.3 ± 2.2	13.5 ± 2.2	0.374	13.4 ± 2.1	13.4 ± 2.3	0.774	13.4 ± 2.2	13.4 ± 2.2	0.953
AV Vmax (m/s)	4.4 ± 0.5	4.4 ± 0.5	4.4 ± 0.6	0.564	4.2 ± 0.6	4.4 ± 0.5	**<0.001**	4.4 ± 0.5	4.4 ± 0.5	0.271
AV MAX (mmHg)	77.7 ± 18.7	77.2 ± 17.7	78.6 ± 20.1	0.407	73.2 ± 18.9	79.0 ± 18.4	**0.002**	76.7 ± 19.1	78.6 ± 18.3	0.215
AV MPG (mmHg)	46.0 ± 11.7	45.6 ± 11.2	46.6 ± 12.5	0.300	43.1 ± 12.6	46.8 ± 11.3	**0.002**	45.0 ± 11.8	46.8 ± 11.5	0.068
TRVmax (m/s)	2.7 ± 0.8	3.2 ± 0.5	2.0 ± 0.7	**<0.001**	2.9 ± 0.8	2.7 ± 0.8	**0.020**	3.3 ± 0.5	2.2 ± 0.7	**<0.001**
sPAP (mmHg)	39.5 ± 17.5	50.0 ± 13.0	22.9 ± 8.6	**<0.001**	44.1 ± 18.2	38.2 ± 17.1	**0.001**	53.0 ± 13.5	27.7 ± 10.8	**0.001**
TAPSE (mm)	21.8 ± 4.7	21.2 ± 4.7	22.7 ± 4.5	**<0.001**	15.9 ± 2.4	23.5 ± 3.7	**<0.001**	19.5 ± 4.1	23.8 ± 4.2	**0.001**
TAPSE/sPAP (mm/mmHg)	0.8 ± 0.8	0.5 ± 0.2	1.3 ± 1.0	**<0.001**	0.5 ± 0.4	0.9 ± 0.8	**<0.001**	0.4 ± 0.1	1.1 ± 0.9	**0.001**
**Median ± IQR**										
Creatinine (mg/dl)	1.0 ± 0.5	1.1 ± 0.5	1.0 ± 0.4	**0.018**	1.2 ± 0.5	1.0 ± 0.5	**<0.001**	1.1 ± 0.5	1.0 ± 0.4	**<0.001**
HB (g/dl)	12.8 ± 2.2	12.6 ± 2.5	13.0 ± 1.9	**0.014**	12.8 ± 2.2	12.7 ± 2.2	0.364	12.6 ± 2.7	12.9 ± 2.0	0.109
CK (U/l)	82.0 ± 67.0	81.0 ± 64.0	85.0 ± 76.0	0.216	78.0 ± 59.0	84.0 ± 69.0	0.391	79.0 ± 62.5	85.0 ± 73.0	0.093
NT-proBNP (pg/ml)	2040.0 ± 3605.6	2483.5 ± 4551.6	1393.0 ± 2016.8	**<0.001**	3336.0 ± 4985.5	1693.0 ± 2922.3	**0.001**	3372.0 ± 5276.3	1.174.0 ± 1949.3	**<0.001**
EuroSCORE II	3.8 ± 3.1	3.9 ± 3.6	3.1 ± 2.4	**<0.001**	5.1 ± 3.6	3.3 ± 2.7	**<0.001**	4.1 ± 3.6	3.1 ± 2.4	**<0.001**

Values are presented as mean ± SD, median ± IQR, or number (%), as appropriate. Comparisons were made using chi-squared test, Student’s t-test, or Mann–Whitney U test. Significant p-values are indicated in bold.

Abbreviations: AF = Atrial Fibrillation; AV MAX = Aortic Valve Maximum Pressure Gradient; AV MPG = Aortic Valve ; Mean Pressure Gradient; AV Vmax = Aortic Valve Maximum Velocity; BSA = Body Surface Area; BMI = Body Mass Index; CHD = Coronary Heart Disease; CK = Creatine Kinase; COPD = Chronic Obstructive Pulmonary Disease; HB = Hemoglobin; HK = Hematocrit; IVSd = Interventricular Septal Diameter in diastole; LVEF = Left Ventricular Ejection Fraction; LVEDD = Left Ventricular End-Diastolic Diameter; MI = Myocardial Infarction; MR = Mitral Valve Regurgitation; NYHA = New York Heart Association Functional Classification; TR = Tricuspid Valve Regurgitation PAOD = Peripheral Arterial Occlusive Disease; sPAP = systolic Pulmonary Artery Pressure; SVi = Stroke Volume Index; TAPSE = Tricuspid Annular Plane Systolic Excursion

### Procedural and early clinical outcomes

Early procedural and in-hospital outcomes are summarized in Table 2. Overall 30-day mortality was low (2.3%) but higher in patients with reduced TAPSE compared with those with preserved TAPSE (4.8% vs. 1.6%, p = 0.037). Major stroke occurred more frequently in patients with impaired RV–PA coupling than in those with preserved coupling (4.2% vs. 1.3%, p = 0.034), whereas no significant differences were observed for elevated sPAP or for other procedural complications. Paravalvular leakage was predominantly none or mild, and no cases of severe PVL were observed.

**Table 2:** Early clinical and procedural outcomes after TAVI stratified by right heart parameters

	Total	sPAP ≥ 35 mmHg			TAPSE ≤ 18 mm			TAPSE/sPAP < 0.55 mm/mmHg		
		Yes	No	p	Yes	No	p	Yes	No	p
**No. (%)**										
Total	565 (100.0)	346 (61.2)	219 (38.8)	-	126 (22.3)	439 (77.7)	-	263 (46.5)	302 (53.5)	-
30d-mortality	13 (2.3)	11 (3.2)	2 (0.9)	0.080	6 (4.8)	7 (1.6)	**0.037**	9 (3.4)	4 (1.3)	0.097
Pacemaker	71 (12.6)	43 (12.4)	28 (12.8)	0.901	14 (11.1)	57 (13.0)	0.576	30 (11.4)	41 (13.6)	0.438
Major Vascular Complications	18 (3.2)	14 (4.0)	4 (1.8)	0.142	4 (3.2)	14 (3.2)	0.995	11 (4.2)	7 (2.3)	0.205
Major Stroke	15 (2.7)	12 (3.5)	3 (1.4)	0.129	3 (2.4)	12 (2.7)	0.825	11 (4.2)	4 (1.3)	**0.034**
No PVL Mild PVL Moderate PVL Severe PVL	154 (27.2) 334 (59.1) 77 (13.7) 0 (0.0)	104 (30.1) 188 (54.3) 54 (15.6) 0 (0.0)	50 (22.8) 146 (66.7) 23 (10.5) 0 (0.0)	0.125 **0.019** 0.174 -	31 (24.6) 72 (57.1) 23 (18.3) 0 (0.0)	123 (28.0) 262 (59.7) 54 (12.3) 0 (0.0)	0.343 0.706 0.078 -	83 (31.6) 137 (52.1) 43 (16.3) 0 (0.0)	71 (23.5) 197 (65.2) 34 (11.3) 0 (0.0)	0.071 **0.007** 0.123 -

Data are presented as n (%). p values refer to comparisons between groups

Abbreviations: PVL = Paravalvular Leakage; sPAP = Systolic Pulmonary Artery Pressure; TAPSE = Tricuspid Annular Plane Systolic Excursion

### Survival analysis according to right heart function

**Fig 1.**
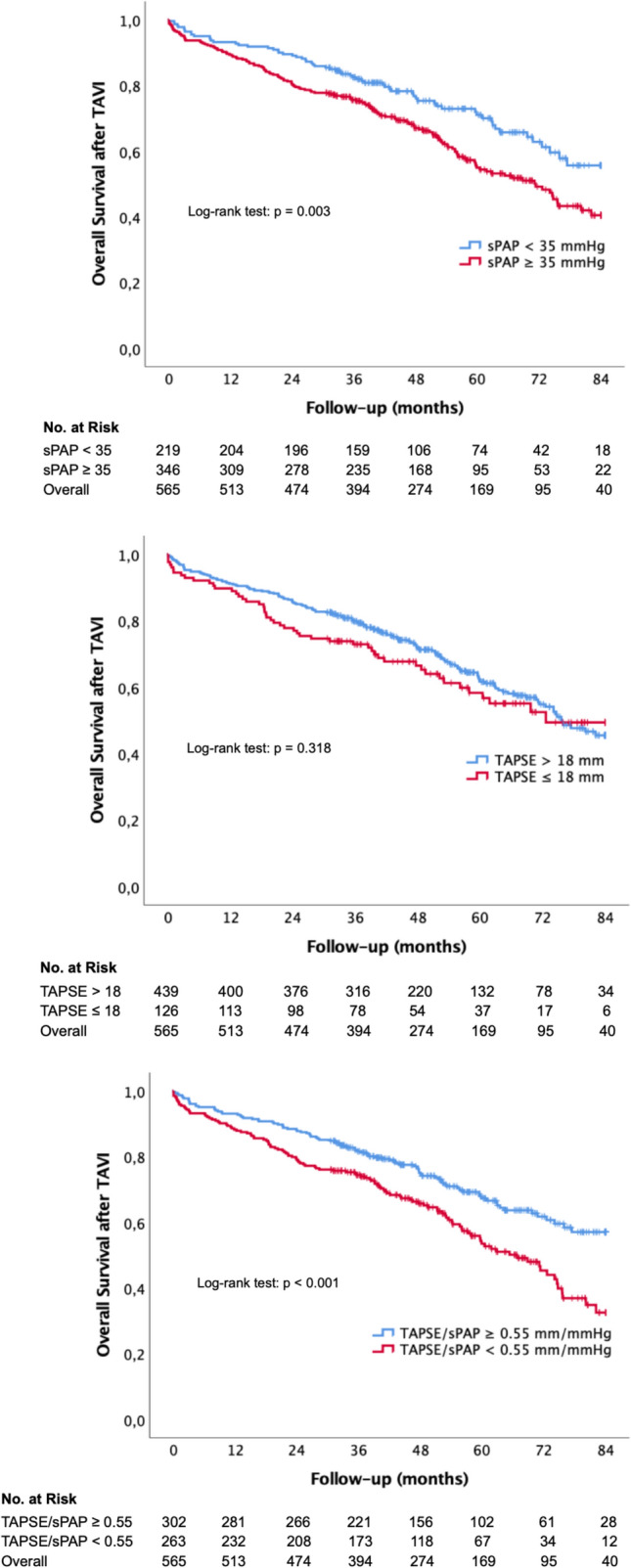
Kaplan–Meier survival curves stratified by **(A)** sPAP (sPAP ≥ 35 mmHg), **(B)** TAPSE (TAPSE ≤ 18 mm), and **(C)** RV–PA coupling (TAPSE/sPAP < 0.55 mm/mmHg). Long-term survival was significantly lower in patients with elevated sPAP (p = 0.004) and impaired TAPSE/sPAP (p < 0.001), while TAPSE alone did not significantly predict mortality (p = 0.318, log-rank test). Abbreviations: No. = Number; sPAP = systolic Pulmonary Artery Pressure; TAPSE = Tricuspid Annular Plane Systolic Excursion

Kaplan–Meier survival analysis of a mean follow-up time of 47.1 ± 22.8 months demonstrated significant differences in long-term mortality based on right heart functional parameters (Fig 1). Patients with elevated sPAP (sPAP ≥ 35 mmHg - Fig 1A) exhibited a significantly reduced overall survival compared to those with lower sPAP values (log-rank p = 0.004). The most pronounced prognostic separation was observed in patients with impaired right ventricular–pulmonary arterial (RV–PA - Fig 1B) coupling, defined as a TAPSE/sPAP ratio < 0.55 mm/mmHg, who experienced markedly higher mortality throughout follow-up (log-rank p < 0.001).

In contrast, reduced right ventricular systolic function (TAPSE ≤ 18 mm - Fig 1C) was not significantly associated with long-term mortality in unadjusted survival analysis (log-rank p = 0.318), despite a numerically higher event rate.

### Univariate cox regression in clinical subgroups

**Fig 2.**
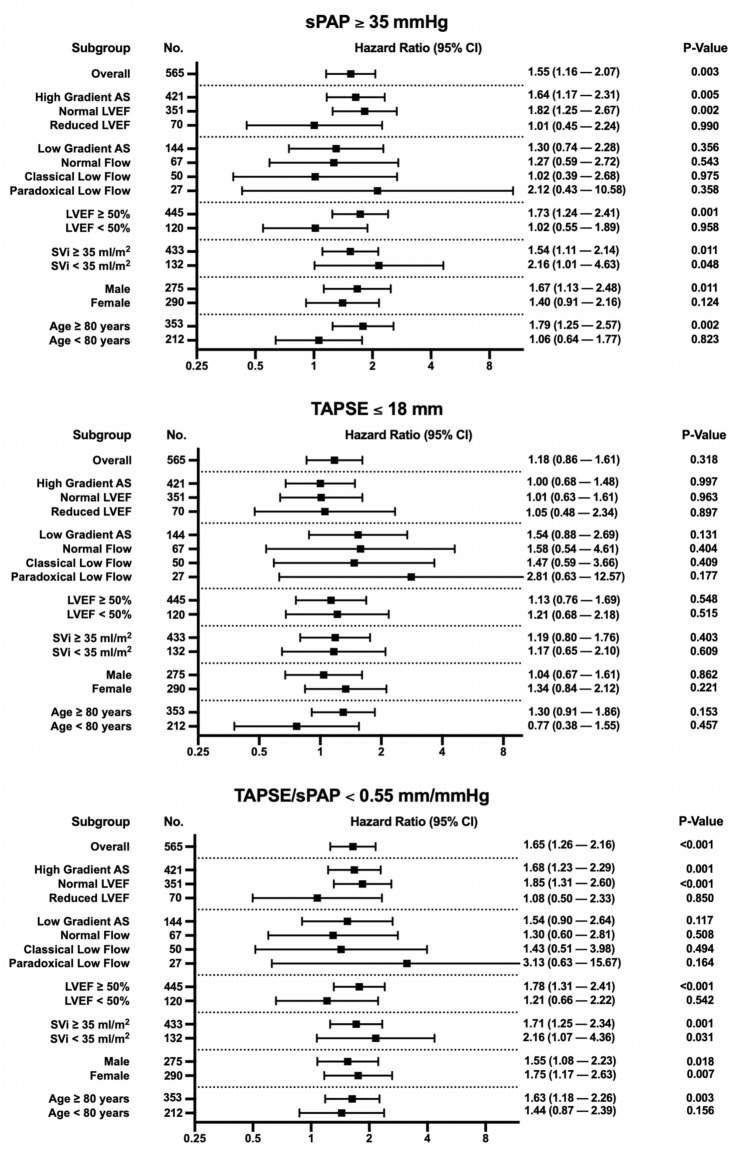
Univariate Cox regression for long-term mortality stratified by clinical subgroups according to right heart parameters: sPAP (sPAP ≥ 35 mmHg), TAPSE (TAPSE ≤ 18 mm), and RV–PA coupling (TAPSE/sPAP < 0.55 mm/mmHg). Subgroups include age (<80 / ≥80 years), sex, LVEF, SVi and aortic stenosis gradient status. Hazard ratios (HR) with 95% confidence intervals (CI) are shown. TAPSE/sPAP consistently demonstrated the strongest and most uniform prognostic value across all subgroups. Abbreviations: AS = aortic stenosis; CI = Confidence Interval; LVEF = Left Ventricular Ejection Fraction; No. = Number; sPAP = systolic Pulmonary Artery Pressure; SVi = Stroke Volume Index; TAPSE = Tricuspid Annular Plane Systolic Excursion

To evaluate the prognostic relevance of right heart parameters across clinical contexts, univariate Cox regression analyses were performed in subgroups defined by age, sex, LVEF, SVi, and AS gradient (Fig 2).

Both elevated sPAP and impaired RV–PA coupling were associated with increased long-term mortality in the overall cohort and across major subgroups. In patients with preserved LVEF, sPAP (HR = 1.55, 95% CI 1.09–2.20) and TAPSE/sPAP (HR = 1.90, 95% CI 1.37–2.63) were significantly associated with mortality, whereas TAPSE was not (HR = 1.28, 95% CI 0.83–1.97). Similar patterns were observed in patients with normal flow (sPAP HR = 1.58; TAPSE/sPAP HR = 1.89) and in high-gradient AS (sPAP HR = 1.51; TAPSE/sPAP HR = 1.82).

Age- and sex-stratified analyses confirmed these findings: TAPSE/sPAP < 0.55 showed the highest hazard ratios in patients aged ≥80 years (HR = 1.82) and in men (HR = 1.93), while TAPSE alone was not associated with mortality in any subgroup.

As these analyses are unadjusted, subsequent interaction and multivariate analyses were performed to account for confounding and effect modification.

### Interaction term analyses

**Fig 3.**
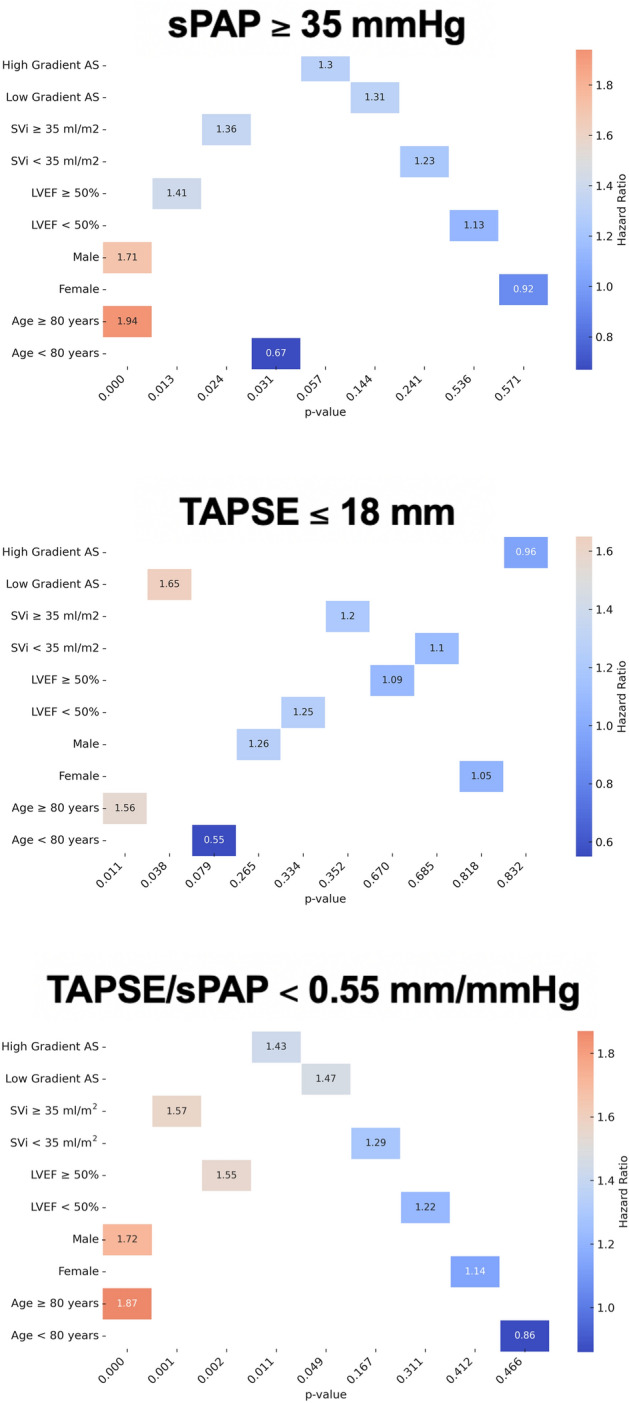
Interaction term analysis for long-term mortality showing the effect modification of clinical subgroups on the prognostic relevance of three right heart parameters: sPAP (sPAP ≥ 35 mmHg), TAPSE (TAPSE ≤ 18 mm), and RV–PA coupling (TAPSE/sPAP < 0.55 mm/mmHg). Subgroups include age, sex, LVEF, SVi and AS gradient type. Abbreviations: AS = aortic stenosis; LVEF = Left Ventricular Ejection Fraction; sPAP = systolic Pulmonary Artery Pressure; SVi = Stroke Volume Index; TAPSE = Tricuspid Annular Plane Systolic Excursion

To assess effect modification, interaction analyses were performed across predefined subgroups (Fig 3 and Supplementary Tables 1–3).

For sPAP ≥ 35 mmHg, the association with mortality was stronger in patients with preserved LVEF (HR 1.41; p = 0.013) and normal SVi (HR 1.36; p = 0.024), and was most pronounced in men (HR 1.71; p < 0.001) and in patients aged ≥80 years (HR 1.94; p < 0.001). No significant associations were observed in patients with reduced LVEF, low flow, or in women.

TAPSE/sPAP < 0.55 showed a highly similar pattern, with significant associations in preserved LVEF (HR 1.55; p = 0.002), normal SVi (HR 1.57; p = 0.001), male sex (HR 1.72; p < 0.001), and age ≥80 years (HR 1.87; p < 0.001), but not in the complementary subgroups.

In contrast, TAPSE ≤ 18 mm showed a weaker and inconsistent profile, with significant associations only in older patients (HR 1.56; p = 0.011) and in those with low-gradient AS (HR 1.65; p = 0.038).

### Multivariate cox regression

In multivariate Cox models adjusted for relevant covariates and predefined subgroups (Table 3), right heart parameters were not independently associated with mortality in patients with high-gradient aortic stenosis or preserved stroke volume (SVi ≥ 35 ml/m^2^).

**Table 3:** Multivariate Cox regression analysis for long-term mortality across clinical subgroups, evaluating the independent prognostic value of sPAP ≥ 35 mmHg, TAPSE ≤ 18 mm, and impaired RV–PA coupling (TAPSE/sPAP < 0.55 mm/mmHg)

Multivariate Cox Regression	sPAP ≥ 35 mmHg	TAPSE ≤ 18 mm	TAPSE/sPAP < 0.55 mm/mmHg
High Gradient AS	0.97 (0.65 — 1.45) p = 0.894	0.62 (0.32 — 1.21) p = 0.160	0.97 (0.63 — 1.51) p = 0.898
SVi ≥ 35 ml/m^2^	1.03 (0.71 — 1.51) p = 0.876	1.17 (0.64 — 2.12) p = 0.616	1.10 (0.74 — 1.66) p = 0.632
Age ≥ 80 years	2.05 (1.4 — 3.00) **p < 0.001**	2.41 (1.15 — 5.05) **p = 0.020**	1.81 (1.18 — 2.79) **p = 0.007**
Sex (male)	1.64 (1.17 — 2.29) **p = 0.004**	1.15 (0.64 — 2.08) p = 0.640	1.47 (1.01 – 2.14) **p = 0.045**
LVEF	1.00 (0.99 – 1.02) p = 0.650	1.01 (0.98 — 1.03) p = 0.558	1.00 (0.98 — 1.02) p = 0.905
EuroSCORE II	1.06 (1.03 — 1.09) **p < 0.001**	1.03 (0.97 — 1.09) p = 0.396	1.04 (1.01 — 1.08) **p = 0.013**

Results are presented as HR with 95% CI and corresponding *p*-values. Statistically significant associations are shown in bold

Abbreviations: AS = aortic stenosis; LVEF = Left Ventricular Ejection Fraction; sPAP = systolic Pulmonary Artery Pressure; SVi = Stroke Volume Index; TAPSE = Tricuspid Annular Plane Systolic Excursion

In contrast, in patients aged ≥80 years, all three parameters independently predicted mortality, including sPAP ≥ 35 mmHg (HR 2.05, p < 0.001), TAPSE ≤ 18 mm (HR 2.41, p = 0.020), and TAPSE/sPAP < 0.55 (HR 1.81, p = 0.007). Male sex was independently associated with mortality in models including sPAP (HR 1.64, p = 0.004) and TAPSE/sPAP (HR 1.47, p = 0.045), whereas TAPSE alone was not predictive. LVEF was not independently associated with mortality, while EuroSCORE II remained an independent predictor in models including sPAP and TAPSE/sPAP.

## Discussion

Our study demonstrates that the prognostic relevance of right heart parameters before TAVI is not uniform, but differs across clinically relevant patient subgroups. While univariate analyses suggested associations between elevated sPAP, impaired RV–PA coupling, and mortality, multivariate models showed that these parameters provided independent prognostic information primarily in patients with limited physiological reserve, most notably elderly patients and men. In contrast, TAPSE alone showed limited and inconsistent prognostic value; although reduced TAPSE was associated with higher early (30-day) mortality, this signal did not translate into a robust association with long-term all-cause mortality.

This observation likely reflects both methodological and pathophysiological factors. TAPSE is a load-dependent parameter that primarily reflects longitudinal RV shortening and may remain preserved in early or pressure-dominant stages of RV dysfunction [5, 6]. In aortic stenosis, RV impairment is frequently driven by progressive afterload increase rather than intrinsic contractile failure, particularly in the pre-procedural setting. Accordingly, integrative indices such as the TAPSE/sPAP ratio more accurately capture RV–pulmonary arterial interaction and prognosis [8, 24]. Differences compared with prior studies, including those demonstrating prognostic value of TAPSE alone, may relate to variations in timing of assessment, case-mix, and endpoints [3, 10].

In contrast to Meucci et al. [24], who reported that post-procedural RV–PA uncoupling and its longitudinal evolution predict long-term mortality, the present study focuses on the pre-procedural phase and identifies vulnerable phenotypes in whom right heart assessment is most clinically informative before intervention. Together, these findings emphasize that outcomes after TAVI are influenced not only by left ventricular function but also by right ventricular vulnerability, underscoring the need for targeted pre-procedural assessment in selected patients.

### Afterload dominates pre-procedural RV vulnerability

Our findings indicate that RV pressure overload is the predominant hemodynamic burden in many patients undergoing TAVI. Chronic transmission of elevated left-sided filling pressures into the pulmonary circulation increases pulmonary artery pressure and RV afterload, thereby challenging RV performance [25]. While sPAP reflects the magnitude of this load, the TAPSE/sPAP ratio provides complementary information by capturing the RV’s functional adaptation to afterload.

In our cohort, elevated sPAP and reduced TAPSE/sPAP characterized a pressure-dominant RV phenotype, but their prognostic relevance was not uniform across all patients. After multivariate adjustment, these parameters were not independently associated with mortality in patients with preserved LVEF, normal SVi, or high-gradient aortic stenosis. In contrast, their prognostic value was most evident in patients with limited physiological reserve, particularly elderly patients and men [26]. Importantly, this pressure-dominant RV dysfunction may occur even in the absence of overt clinical right heart failure and may therefore remain unrecognized during routine assessment.

RV–PA coupling, best reflected by the TAPSE/sPAP ratio, integrates RV contractile function and afterload and thus provides a more sensitive marker of maladaptive RV response than TAPSE alone [27]. In our study, impaired RV–PA coupling was consistently associated with adverse outcomes, especially in patients with preserved LV function, suggesting that afterload-related RV stress rather than intrinsic RV systolic dysfunction is the dominant mechanism in this setting [28].

Subtle abnormalities in pulmonary vascular load and compliance further contribute to RV vulnerability and are not captured by standard anatomical measures [29]. Experimental and clinical data indicate that reduced pulmonary arterial compliance and increased elastance disrupt RV–PA coupling and worsen prognosis [30], which is consistent with the vulnerability observed in our cohort.

Clinically, these findings identify a high-risk but often under-recognized group of TAVI candidates: patients with preserved LVEF and flow but elevated pulmonary pressures and impaired RV–PA coupling. In such patients, delayed intervention may permit progression toward irreversible RV maladaptation. Incorporating RV afterload and RV–PA coupling into pre-procedural assessment may help identify a vulnerable pre-procedural phenotype [4]; however, the implications for earlier referral or optimized timing of TAVI remain hypothesis-generating, as the present study was limited to single-point pre-procedural assessment without longitudinal evaluation of RV recovery or waiting-time effects.

### Age as a determinant of right heart vulnerability

Aging exerts a profound yet under-recognized impact on the RV. In our study, age ≥80 years significantly amplified the prognostic relevance of elevated sPAP and reduced TAPSE/sPAP. This likely reflects age-related myocardial changes, including increased fibrosis, reduced capillary density, impaired mitochondrial function, and diminished β-adrenergic responsiveness, which together limit myocardial reserve [31, 32]. As a result, the RV, which is intrinsically less tolerant of pressure overload, becomes particularly vulnerable to rising afterload [2]. Importantly, myocardial fibrosis was not directly assessed in the present study; therefore, these mechanisms are discussed as pathophysiological concepts based on prior experimental and imaging literature rather than direct tissue characterization in our cohort.

In older adults, diastolic dysfunction, atrial fibrillation, and vascular stiffening lead to chronically elevated LV filling pressures, progressive pulmonary venous hypertension, and increasing RV afterload [33]. This process disrupts RV–PA coupling and often evolves insidiously [34], while conventional markers such as TAPSE may remain preserved in early stages. Because RV–PA uncoupling in older individuals tends to occur earlier and at lower sPAP levels, reflecting reduced physiological reserve or “RV frailty,” the TAPSE/sPAP ratio emerges as a more sensitive marker of early maladaptation [27].

These observations have direct clinical implications. Current TAVI risk scores do not account for RV function or age-specific RV vulnerability. Incorporating TAPSE/sPAP into pre-procedural assessment, particularly in elderly patients, may improve detection of occult RV strain and identify individuals at higher procedural and long-term risk. Age-adapted thresholds may further refine risk stratification. Moreover, elderly patients with pre-procedural RV–PA uncoupling may benefit from closer post-TAVI surveillance, including serial echocardiographic assessment to guide follow-up intensity and medical management.

### Male sex as a distinctive right heart failure phenotype

In our cohort, elevated sPAP and impaired RV–PA coupling were more strongly associated with mortality in men than in women, suggesting that adverse right heart phenotypes carry greater prognostic weight in male patients [35]. Sex-related differences in cardiovascular adaptation are increasingly recognized, but their relevance for right heart vulnerability in the setting of TAVI remains incompletely understood.

Several biological mechanisms have been proposed that may contribute to these differences. Estrogen exerts cardioprotective effects relevant to right heart physiology, including improved mitochondrial efficiency, enhanced nitric oxide bioavailability, and reduced myocardial fibrosis [36–40]. In contrast, testosterone has been associated with profibrotic remodeling, oxidative stress, and reduced vascular compliance, which may impair RV adaptation to chronic pressure load [36, 41–43]. Together, these mechanisms may partly explain why male patients appear more vulnerable to pressure-induced RV dysfunction.

These molecular differences are reflected at the clinical level. Men undergoing TAVI more frequently exhibit higher left ventricular mass and increased vascular stiffness [44, 45], both of which augment pulmonary vascular load and RV afterload. However, current risk models do not incorporate sex-specific modifiers, and identical values of RV indices may therefore carry different prognostic implications in men and women.

From a clinical perspective, male sex appears to act as a risk amplifier in the presence of elevated RV afterload and impaired RV–PA coupling. While our data do not support sex-specific treatment strategies, they highlight the need for heightened awareness and potentially closer follow-up in male patients with adverse right heart profiles.

More broadly, our findings support the concept that sex should be considered a biological variable in RV-focused risk assessment. Future studies should evaluate whether sex-stratified thresholds improve risk prediction and whether targeted interventions can preserve RV reserve in high-risk male patients undergoing TAVI.

## Limitation

This study has several limitations. Its retrospective single-center design limits causal inference and generalizability. Right heart parameters were derived from TTE and are therefore subject to measurement variability, particularly for echocardiographic estimation of sPAP, which may be less accurate in the presence of very mild or severe TR or suboptimal Doppler signal quality. Although inter-observer variability was assessed in a representative subset, variability inherent to echocardiographic measurements cannot be entirely excluded.

TAPSE and TAPSE/sPAP, while guideline-recommended and widely used, reflect predominantly longitudinal right ventricular shortening and represent simplified surrogates of complex RV mechanics. Accordingly, these parameters may not fully capture global RV systolic function in all clinical scenarios. The use of fixed cut-off values improves clinical interpretability but may oversimplify continuous or non-linear risk relationships, and residual confounding cannot be excluded.

Subgroup and interaction analyses were exploratory and require confirmation in prospective studies. Furthermore, cause-specific mortality and heart failure–related hospitalizations could not be reliably adjudicated due to the retrospective design and limited availability of detailed event data; therefore, analyses were restricted to all-cause mortality. Finally, invasive hemodynamic assessment and advanced imaging modalities, including RV strain and myocardial tissue characterization by cardiac magnetic resonance or dedicated computed tomography protocols, were not routinely available, and evolving TAVI techniques and expanding indications during the study period may limit applicability to contemporary lower-risk and younger populations.

## Conclusion

This study demonstrates that right heart dysfunction — particularly increased RV afterload and impaired RV–pulmonary arterial coupling — is a major determinant of long-term outcome after TAVI and provides prognostic information beyond conventional left heart–focused assessment. RV–PA coupling, quantified by the TAPSE/sPAP ratio, outperforms isolated measures of RV systolic function and identifies high-risk patients, particularly those with preserved left heart function, normal flow, elevated RV afterload, advanced age, and male sex.

These findings support the integration of right heart assessment into routine pre-procedural evaluation and heart team decision-making and may help improve risk stratification, procedural timing, and follow-up strategies in patients undergoing TAVI.

## Data Availability

Data underlying this study are available from the corresponding author upon reasonable request.

## Abbreviations

AS: Aortic Stenosis
LVEF: Left Ventricular Ejection Fraction
PA: Pulmonary Artery
pts.: patients
RV: Right Ventricle
sPAP: systolic Pulmonary Artery Pressure
SVi: Stroke Volume Index
TAPSE: Tricuspid Annular Plane Systolic Excursion
TAVI: Transcatheter Aortic Valve Implantation
